# The work Lifestyle-integrated Functional Exercise program for preventing functional decline in employees aged 55 years and older: development and initial evaluation

**DOI:** 10.1186/s11556-024-00356-5

**Published:** 2024-08-06

**Authors:** Yvonne Ritter, Diana Pfister, Greta M. Steckhan, Susanne Voelter-Mahlknecht, Britta Weber, Rolf Ellegast, Christian Koch, Frank Bausch, Markus Gruber, Michael Schwenk

**Affiliations:** 1 Human Performance Research Centre, Department of Sport Science, University of Konstanz, Universitätsstraße 10, Constance, 78464 Germany; 2 Institute of Occupational Medicine, Charité-Universitätsmedizin Berlin, Corporate Member of Freie Universität Berlin and Humboldt Universität Zu Berlin, Augustenburger Platz 1, Berlin, 13353 Germany; 3 Institute for Occupational Health and Safety of the German Social Accident Insurance (IFA), Sankt Augustin, Germany; 4 Managing Business Analyst, Capgemini, Cologne, Germany

**Keywords:** Occupational health, Working environment, Aging, Exercise, Prevention, Functional decline, Older employees

## Abstract

**Background:**

Despite the global increase in older employees, workplace physical activity interventions (WPAIs) for this target group have not yet been sufficiently developed. The major drawback of existing WPAIs is low adherence due to lack of time or limited motivation. A novel approach could be to integrate tailored neuromotor and strength exercises into everyday working tasks to prevent the functional decline of older employees at the workplace without needing much additional time for training. This approach was tested in the present study by evaluating the proof-of-concept of a novel WPAI based on the Lifestyle-integrated Functional Exercise (LiFE) program integrated into a working environment (wLiFE55 +).

**Methods:**

The proof-of-concept of wLiFE55 + was quantified within a 4-week pre-post exercise intervention study by measuring (1) feasibility including adherence, activity frequency, adverse events and acceptance (integrability of wLiFE55 + activities, perceived improvement and safety, satisfaction, physical demand, personal trainer session, intervention content) and (2) pre-to-post changes in neuromotor function (12-Level Balance Scale, 12-LBS; Community Balance and Mobility Scale, CBM), strength (60sec Chair Stand Test), and PA (1-week activity monitoring). For statistical analysis, the median and interquartile range (IQR) were computed. For pre-to-post changes, Wilcoxon signed-rank tests with effect size (r) were also performed.

**Results:**

Seventeen older employees (mean age 59 years, 8 female) were included of which fifteen completed the study. The intervention adherence was 100%, and the activity adherence was 58% (9 out of 12 maximum possible wLiFE55 + activities implemented). Depending on the specific activity, the frequency of practice ranged between 25–75% of the days of the intervention period, and single wLiFE55 + activities were practiced between one and three times per day. No adverse events occurred, and acceptance was high. Pre-to-post increases with medium effect sizes were found for neuromotor function (CBM, 12-LBS) and specific PA variables (total sedentary time, sedentary bouts > 30 min).

**Conclusion:**

The results of the study highlight the feasibility of wLiFE55 + in a work setting with older employees. The pre-to-post increases observed in neuromotor measures and reductions in sedentary time suggest that wLiFE55 + may counteract the age-related functional decline in older employees and justifies future studies in this field. The next steps are program adjustments to boost exercise frequency and evaluating wLiFE55 + in a randomized controlled trial.

**Supplementary Information:**

The online version contains supplementary material available at 10.1186/s11556-024-00356-5.

## Background

Population aging and older retirement age mean that more older employees (i.e., those aged 55 years and older [1, 2]) will remain in the workforce [3, 4]. An aging population means an aging workforce, making it essential for companies to recruit and retain older employees to meet their personnel requirements [5]. However, older employees face an age-related functional decline, and their physical work capacity is up to 50% lower [6]. Decreasing physical capacities include muscular strength and endurance [7] and neuromotor function (balance, agility, flexibility and coordination) [8–11].

Physical inactivity contributes to age-related functional decline [12, 13]. Without sufficient training, muscular strength decreases by approximately 10–15% per decade [14]. Maintaining muscle strength is important for health, as it is positively associated with metabolic function [15], and is fundamental for carrying out daily activities [16]. Neuromotor function decreases by 10% per decade [17], which can result in mobility impairments and falls. Neuromotor function is also positively associated with neurocognitive health [18].

Physical activity (PA) is a powerful tool for preventing age-related functional decline and maintaining strength and neuromotor function [19]. However, only 23% of adults comply with PA guidelines, and compliance continues to decline with age [20]. It is therefore particularly important to encourage people to stay physically active as they grow older. This also applies to PA at work. Therefore, companies must offer workplace physical activity interventions (WPAI) for older employees. The WPAI for older employees needs to specifically address the domains of strength and neuromotor function to counteract age-related decline [21]. A systematic review reported only a small number of WPAI for older employees. Most of these interventions focused on aerobic exercise. The authors explicitly called for the development of programs with strength and neuromotor training. Furthermore, all WPAIs were carried out in addition to work. These additional interventions require employees to take extra time outside their regular tasks, i.e. in exercise courses. However, these interventions are generally not seamlessly integrated into the work environment and are perceived as optional add-ons. Therefore, these treatments may be ineffective in the long run due to low adherence [22]. To maximize adherence, new WPAI tools to motivate older adults to adopt more physically active lifestyles in the workplace are needed.

One highly promising alternative approach is the *Lifestyle-integrated Functional Exercise (LiFE)* program, which integrates specific exercises for improving strength and neuromotor function into everyday activities while considering behavioral change to foster long-term adherence through habit formation [23, 24]. LiFE integrates short exercise bouts into everyday life for older adults (60 +), making it feasible without requiring additional time for an exercise program [25]. Previous studies [22] have shown successful integration with greater adherence to LiFE (64% of participants) than to additional training (53%) due to the high degree of ‘personalization’ of the integrated activities. A recent review paper [25] shows evidence that LiFE improves the physical capacity of various target populations. The most relevant studies for the present topic are related to the adapted aLiFE for young retired seniors [19, 24, 26–28]. The exercises as well as the behavioral change techniques have been adjusted for this younger target population [24]. Studies have demonstrated the feasibility of aLiFE [19, 26, 27] and its effects on specific physical capacity measures such as balance and mobility [24] and the use of technology [27]. However, to the best of our knowledge, aLiFE has not been transferred to a workplace setting yet.

Previous WPAI studies of older employees have not focused on integrating exercise into workplace routines [29]. Work commonly occupies almost half of the waking hours of older employees and therefore provides a large ‘time bank’ for offering multiple opportunities to integrate exercises into daily routines at the workplace [30]. Integrating PA to improve strength and neuromotor function in the work environment could be a promising strategy for employees in an office setting, as this group has little PA during work and could therefore particularly benefit from such training. The primary objectives of this study were 1) to develop a wLiFE55 + intervention by adapting aLiFE for the working environment and for a target group of older employees and 2) to assess the feasibility of the newly developed wLiFE55 + program. The secondary objectives included exploring pre-post-intervention changes in neuromotor function, strength, and PA patterns after 4 weeks of wLiFE55 + training.

## Methods

The development and feasibility testing of the wLiFE55 + program were based on the framework for adapting public health interventions [31]. Stage 1 involved (a) community assessment, (b) expert consultation, and (c) adaptation of aLiFE to develop wLiFE55 +. Stage 2 included feasibility testing of wLiFE55 + in a pilot study.

### Stage 1: development of the wLiFE55 + program

#### (a) Community assessment

Community assessment included (i) assessing organizational capacity to implement the program and (ii) need assessment. (i) To assess the organizational capacity required to implement the program and how recruitment should proceed, a meeting was held with the person responsible for the sports program (PB) and the person responsible for occupational health management (KM) at the University of Konstanz. The organization (University of Konstanz) expressed the capacity and need for wLiFE55 + and supported participant recruitment using flyers, mailing lists (newsletter), and a webpage. (ii) For need assessments, the first author (YR) and second author (DP) carried out literature research in the field of WPAI [32–34] and identified environmental and situational cues for integrating the wLiFE55 + activities into an office environment (see Table 1, appendix). The results of the need assessment were incorporated in stage (c).

**Table 1:** Adaptation from aLiFE to wLiFE55 + in new situations in the working environment for specific wLiFE55 + activities

Domain	Activity	Situation in the working environment		
		**On the way to work/at home**	**In the office**	**Break**
**Neuromotor function**	*Tandem stand*	Do the *tandem stand* when you wait for the bus	Do the *tandem stand* when you are booting your computer	Do the *tandem stand when you* wait for the lunch in the mensa
	*Tandem walk*	Do the *tandem walk* when you walk the last few meters to your office/ home	Do the *tandem walk* when you take out documents from a work cabinet	Do the *tandem walk* when you go to the coffee machine
	*One-leg stand*	Do the *one-leg stand* when you tie your shoes	Do the *one-leg stand* at a height adjustable desk when writing mails	Do the *one-leg stand* when you wait for the coffee machine
	*Leaning forward and backward*	Do the *leaning forward and backward* before opening the entrance door to the office	Do the *leaning forward and backward* when you get an overview over your tasks and meetings for the day	Do the *leaning forward and backward* when you warm up your lunch in the microwave
	*Stepping over objects*	Do the *stepping over objects* above any curbside	Do the *stepping over* a folder before using it	Do the *stepping over objects* above any doorstep
	*Stepping and changing direction*	Do the s*tepping and changing direction* when you walk through the building to leave work	Do the s*tepping and changing direction* when you make a phone call	Do the s*tepping and changing direction* when you take a walk outside during the break
	*Stepping, hopping and jumping in different ways*	Do the s*tepping, hopping and jumping in different ways* when you enter the office corridor	Do the s*tepping, hopping and jumping in different ways* when you want to leave the office	Do the s*tepping, hopping and jumping in different ways* when you enter the hallway on your way to the office kitchen
**Strength**	*Squat/ one-legged squatting*	Do the *squatting* when you put down your working bag	Do the *squatting* when you do phone calls with colleagues	Do the *squatting* when you remove a cup
	*Lunging*	Do the *lunging* when you remove objects from your working bag	Do the *lunging* when you wait at the copy machine or on the way to the printer	Do the *lunging* when you throw something in the bin
	*Sit-to-stand*	Do the *sit-to-stand* when passing a park bench	Do the *sit-to-stand* when receiving mails or phone calls	Do the *sit-to-stand* when you look at the clock
	*Toe standing/walking*	Do the *toe walking* when you enter your office	Do the *toe standing or walking* when you talk with colleagues	Do the *toe standing or walking* when you pick up objects from a high level
	*Heel standing/walking*	Do the *heel walking* when you enter your home	Do *heel standing* when you stand at your working desk	Do the *heel standing or walking* when you pick up objects from the floor
	*Stair climbing*	Do the *stair climbing* when going to your office	Do the *stair climbing* when walking to the office	Do the *stair climbing* when you go to the mensa
	*Move leg sideways, when lying, standing, walking*	Do the *move leg sideways* when a walking to your office	Do the *move leg sideways* when a colleague passes your office	Do the *move leg sideways* when you air your office
	*Tigthening muscles*	Do the *tightening muscles* when you wait for the bus	Do the *tightening muscles* when you finished a working task	Do the *tightening muscles* when you finished eating
**PA**	*Walk longer*	*Walk longer* to the office	*Walk longer* when you are going to the office of a colleague	*Walk longer* back to the office after lunch
	*Walk faster*	*Walk fast* for 100 m when you are on the way to the office	*Walk faster* when you go to the restroom/ meeting	*Walk faster* when you are going to the coffee machine
	*Sit less*	*Sit less* on the way from work home and get on the bus one stop later	*Stand up* after 60 min of sitting	*Sit less* during your lunch break and take a walk
	*Break up sitting*	Do the *break up sitting *and ride a bike instead of a driving to work once a week	Do the *break up sitting* in your office when checked your calendar	Do the *break up sitting* after lunch

#### (b) Expert consultation

Experts from sports science (MS, YR, DP), occupational health (SVM, GMS), occupational safety (RE, BW), and the occupational healthcare industry (FB, CK, BH) were consulted to discuss the adaptations for transferring the aLiFE concept to the new setting and target population. During joint meetings, it was discussed how the (i) aLiFE concept can be transferred to an office setting, (ii) what environmental (e.g., staple documents, set aside bin) and situational (e.g., enter the date in the calendar, phone call with colleague cues are relevant for incorporating wLiFE55 + activities into an office setting, and (iii) how to achieve an adequate training intensity to fulfill the ACSM criteria [9]. The experts decided that the aLiFE activities, designed for retired people (aged 60 +), are sufficiently challenging for older employees (aged 55 +) and meet the ACSM criteria for strength and balance exercises in terms of task challenge and progression. The exercises from aLiFE were retained for wLiFE55 +. In contrast, the environmental and situational cues had to be adapted because aLiFE has not been designed for a workplace setting. However, the behavioral change concept of aLiFE was retained [19].

#### (c) Adaptation of aLiFE to develop wLiFE55+ 

aLiFE is a variant of the LiFE program and has been specially developed for younger older people after retirement [24]. aLiFE served as the basis for the development of wLiFE55 +. aLiFE has been designed based on the ACSM guidelines for neuromotor function and strength training [9]. The aLiFE program targets age-related functional decline related to strength and neuromotor function by integrating personalized activities into daily routines [22] using established behavior change techniques [19]. The aLiFE activities were retained for wLiFE55 + as they have sufficient task challenge for older employees. However, because aLiFE was not developed as a WPAI, the program lacks the corresponding environmental and situational cues needed to integrate activities into daily routines at the workplace. These were added in the course of the adaptation (Table 1). For each activity, the participant manual was supplemented with information about situations offering opportunities for integration exercises into the working environment (Table 1). Based on the environmental and situational cues, the wLiFE55 + activities were subdivided into three categories: 1) on the way to work/ on the way home, 2) in the office, and 3) during the break.

### Stage 2: feasibility pilot study

#### Study design and setting

A pilot study conducted at the University of Konstanz from March to July 2022 aimed to test the feasibility of wLiFE55 +. This study included a 4-week intervention with a pre-to-post design involving four personal trainer sessions at the workplace.

#### Study population

**Fig. 1:**
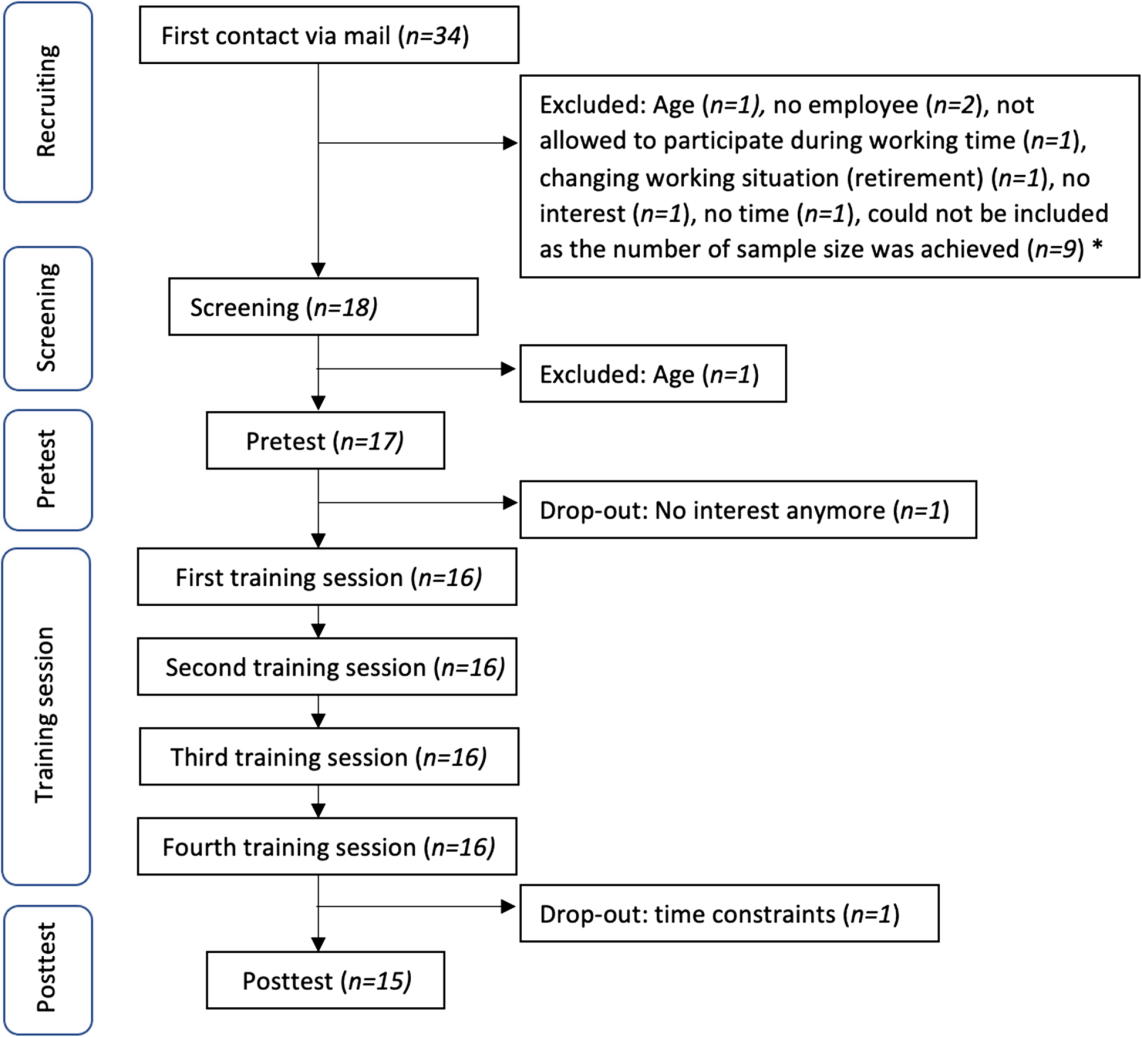
Study design: First contact, screening, pretest, training sessions and posttest. * The number of required participants (*n* = 17) for a pilot study was achieved

A convenient sample of 34 employees was recruited via flyer. The inclusion criteria were a minimum age of 55 years and current employment status. The exclusion criteria were having such diseases where exercise was contraindicated, attending organized exercise classes more than twice a week or exercising more than two hours each week. The participants provided written informed consent that was approved by the ethics committee of the University of Konstanz (ethical approval number: IRB23KN07-005w, date of approval: 12 July 2023). The study design is presented in Fig. 1. Eight participants were not eligible due to these inclusion and exclusion criterias.

#### Study procedures

##### Assessments

The pretest and post-assessments were conducted at the University of Konstanz, except for the ambulatory daily PA assessment. All measurements were validated and selected for the wLiFE55 + target group.

##### Administering the intervention

After the pretest, participants received the wLiFE55 + manual (see participant manual summary in the Supplementary file 1) and met a personal trainer (holding sports science degrees) for a 1.5-h session (see personal trainer session checklist in the Supplementary file 2). During this first session, the personal trainer (a) introduced the wLiFE55 + trainer by the use of the wLiFE55 + manual, (b) evaluated the ability and opportunities for wLiFE55 + activities using the Daily Routine Chart (see Supplementary file 3), (c) assessed the level of difficulty for each activity using the wLiFE55 + Assessment Tool (wLAT55 +) (see Supplementary file 4), (d) discussed goal setting about short- and long term fitness goals, (e) implemented up to four activites (neuromotor function, strength, and PA) linked to specific environmental and situational cues in the working environment, such as working tasks, situations or places, using the Daily Routine Chart (see Supplementary file 3) and the Activity Planner (see Supplementary file 5).

In the 2nd, 3rd and 4th training sessions, the personal trainer (a) clarified any questions related to the wLiFE55 + program, (b) reviewed the wLiFE55 + activities commenced previously, the Activity Planner, (c) taught ways of making the program more effective (upgrading wLiFE55 + activities), (d) implemented up to four wLiFE55 + activities and (e) developed plans for embedding the wLiFE55 + activities into the daily routine. Personal trainers motivated participants and supported adjusting wLiFE55 + activities over three follow-up sessions based on preferences. wLiFE55 + activities were documented in the Activity Planner with details on how, when, and where to perform them, aligning with HAPA (Health Action Process Approach) model recommendations, specifically adjusted to the LiFE concept [19]. The HAPA model served to enrich habit formation theory because of its emphasis on motivational and volitional factors during behavior change. For the planning procedure, participants used implementation intentions [35] by writing the environmental and situational cue followed by the wLiFE55 + activity in if–then sentences (e.g., “If I turn on my computer, then I do the tandem stand”). The integration of behavior change techniques including HAPA into the LiFE program is explained in Boulton, Hawley-Hague [19] and was also used in wLiFE55 + . Intrinsic motivation is another beneficial factor for long-term maintenance PA behavior [36]. Therefore, wLiFE55 + fulfills three psychological needs (autonomy, competence, and connectedness) by empowering participants to independently manage their training and become their own LiFE trainers [37].

#### Measurements

##### Participant characteristics

For the descriptive data, a self-developed questionnaire asked for personal data such as weight, height, education, profession, current working situation, and job satisfaction.

##### Feasibility measures obtained during intervention:

- Adherence: Drop-out rate, intervention adherence and activity adherence.
  o Drop-out rate: Percentage of those who dropped out of the intervention.
  o Intervention adherence: Number of participants in the personal trainer sessions.
  o Activity adherence: Number of wLiFE55 + activities integrated during the intervention in daily life. During each session, a maximum of 4 wLiFE55 + activities could be integrated, for a total of 16.
- Frequency of practice: Number of wLiFE55 + activities per day and percentage of days for each activity reported by the participant in the ‘Activity Planner‘.
  o Daily frequency practice: For each activity, frequency per day, the data were summed and divided through all days the participants executed the activity.
  o Weekly percentage of practice days during the intervention: For each activity, the percentage of days in which the activity was performed was divided by the total days in the intervention’s duration.
- Muscle soreness: pain in the muscles after training
- Adverse events: included self-reported pain and accidents during the intervention.

##### Feasibility measures obtained post-intervention:

- Acceptability of wLiFE55 + activities: defined as integrability, perceived improvement, satisfaction with progress, perceived safety, and physical demands of wLiFE55 + activities using a 6-point Likert scale. Each question was followed by an open-ended question to explain the given ratings.
- Acceptability of the wLiFE55 + program: overall reported acceptability, using a 6-point Likert-scale. Each question was followed by an open-ended question to explain the given ratings.
- Activity preferences: three favorite wLiFE55 + activities.

##### Exploratory pre-post measures of subjective assessments:

The Activities-Specific Balance Confidence-Scale (ABC-D) [38] was used to assess fall-associated self-efficacy. The normal and complex activity scores were calculated. The International Physical Activity Questionnaire (IPAQ) measures movement behavior and categorizes it into different activities, such as profession, transport, household, and leisure time [39]. We used the IPAQ to calculate the walking activity time, moderate activity time, vigorous activity time, and total PA activity time.

##### Exploratory pre-post-measures of objective assessment:

**Neuromotor function**: We used the 12-Level-Balance Scale (12-LBS) to asses static balance. It is an extended version of the 8-Level Balance Scale (8-LBS) [23]. The 12-LBS is a series of static standing positions with increasing difficulty, which is achieved by a) a reduction in the support area, b) sensory handicaps (eyes closed), and c) additional cognitive tasks (see Supplementary file 6). The participants had to complete a balance task for 30 s without support or to perform a reactive step or arm movement before progressing to the next task. The highest balance test performed successfully was rated (maximum score: 12 points). Similarly, the Community Balance Mobility Scale (CBM) was used to assess balance and mobility through 13 items, including unilateral stance, tandem walking, 180° tandem pivot, lateral foot scooting, hopping forward, crouch and walk, lateral dodging, walking and looking, running with controlled stop, forward to backward walking, walk, look and carry, descending stairs, and step-up 1 × step [40]. Except for unilateral stance, all CBM items evaluate dynamic balance. Each item was rated based on standardized instructions and scoring guidelines (range 0–5 points), with a maximum possible score of 96 points.

**Strength**: The 60sec Chair Stand Test (60CST) was utilized to assess lower extremity strength by measuring the number of times participants could stand up and sit down on a chair (45 cm height) without arms within 60 s [41]. For this purpose, the total number of repetitions is presented. To avoid ceiling effects, we chose the 12-LBS, CBM, and 60CST as measuring instruments to challenge the higher performance level of older employees.

**PA**: PA was monitored using the Move 3 accelerometer (move III, movisens GmbH, Karlsruhe, Germany) [42], which was worn on the high wrist. The sensor wear time was recorded, and the PA was categorized before the first and after the fourth personal trainer session. The sensor was removed during water immersion and overnight periods. PA variables were computed with DataAnalyzer (Movisens GmBH, Karlsruhe, Germany, version 1.13.7) and R (software package, version 2023.06.0 + 421). The sensor wear time encompassed the total minutes worn daily (from waking to bedtime). The following PA variables were categorized: activity-related (standing time, PA time, sit-to-stand transfers, steps) and sedentary-related (sedentary time, sedentary bouts > 20 min, > 30 min, and > 60 min and their counts).

**Activity-related PA variables**: The standing time was extracted from activity class 9 (standing) in DataAnalyzer, and the daily minutes were totaled. PA activity time encompassed the sum of activity classes 4 (slope up), 5 (jogging), 6 (slope down), and 7 (walking) from DataAnalyzer, with daily PA minutes. The step count was derived from the DataAnalyzer daily data. Sit-to-stand transfers were calculated when transitioning from activity class 8 (sitting) to either 9 (standing) or 2 (PA).

**Sedentary-related PA variables**: Sedentary time was classified as activity class 8 (sitting/lying), and sedentary bouts were calculated and separated into bouts of > 20 min, > 30 min and > 60 min. The total time and the number of each sedentary bout were calculated.

### Statistical analysis

Participant characteristics were reported using measures of central tendency (mean, median) and dispersion (SD = *standard deviation, IQR* = *interquartile range*), and were analyzed with the JASP Team (2023) (version 0.16.3). Pre-to-post changes were explored using the Wilcoxon signed-rank test because the data was not normally distributed. The effect size (r) for the Wilcoxon test is given by the match rank biserial correlation. The effect size (r) is calculated as the Z statistic divided by the square root of the sample size (N) (Z$\sqrt N$) [43]. The effects were interpreted as small (*r* = 0.1), medium (*r* = 0.3), or large (*r* = 0.5). A p-value less than 0.05 was considered significant, and a p-value greater than 0.05 was considered not significant. Additionally, a subanalysis was performed for low functioning participants for pre-to-post changes based on the median split.

## Results

Thirty-four older employees were screened for eligibility, and 17 were included (Fig. 1). Two participants dropped out after the baseline assessment, reportedly because of no interest or lack of time. No outcome analyses were conducted for these two participants. No adverse events were reported in the study. Fifteen participants completed the study and were included in the analysis.

### Descriptive data

The sample comprised older employees aged 55–68 years (Table 2). Baseline balance and mobility function were high (CBM score 8 points above the reference value [44]). Strength, as measured by the 60CST, ranged between 30 and 63 repetitions. Sedentary behavior (620 min/day) was greater than that reported in a literature review (approximately 600 min/day) [45]. The baseline steps (9262 steps per day) were in the range of a “somewhat active” (7500–9999 steps/day) group [46].

**Table 2:** Baseline characteristics of the study participants (*n* = 17)

Variable	*n* = 17
Age, years	59 ± 4 (55–68)
Women, number	10 (59%)
BMI, kg/m^2^	26 ± 4 (21–36)
60CST, number of repetitions	43 ± 11 (30–63)
CBM (0–96), total score	81 ± 12 (52–99)
Sedentary behaviour, min/day	620 ± 87 (427–773)
PA time, min/day	108 ± 25 (74–173)
Steps, numbers of steps/day	9262 ± 2568 (6095–16038)

The data are presented as the mean ± SD (range) or n (%). *BMI* Body Mass Index, *CBM* Community Balance and Mobility Scale, *60CST* 60sec Chair Stand Test

On average, the participants had a high level of education, and most (80%) were permanently employed. The majority (67%) worked in flextime. The participants worked in the following professional fields: technical professions (*n* = 5), administrative professions (*n* = 9), and healthcare professions (*n* = 3). On average, they worked 34.1 (± 9.4) hours per week and spend 3.6 (± 1.5) days in the office and 1.4 (± 1.5) days working from home. Nine participants worked in a single office, and eight participants worked in a two-person office or multiroom office. On average, participants tended to be satisfied with their job, with few having concerns about job change (see Supplementary file 7).

### Adherence

All participants included in the analysis (*n* = 15) showed 100% intervention adherence. Of a maximum possible 16 wLiFE55 + activities, participants carried out an average of 9 wLiFE55 + activities (± 1.5, range 7–12) during the intervention, corresponding to a mean activity adherence of 57% (range: 44–75%). wLiFE55 + strength activities were most frequently implemented (mean = 3.7 ± 1, range 2–5), followed by wLiFE55 + neuromotor activities (mean = 3.3 ± 0.7, range 2–5) and PA (mean = 2.2 ± 0.7, range 1–3).

### Frequency of practice

Participants included in the analysis (*n* = 15) most frequently implemented *lunging* for the strength module, *one-leg stand* and *tandem walk* for the neuromotor module, and *walking faster* for the PA module, while least frequently implementing *toe and heel standing and walking*, *stepping and changing direction*, and *sitting less* (Table 3).

**Table 3:** wLiFE55 + activities implemented during the intervention (*n* = 15)

Activity module	Frequency of Practice		
	**Activity type (No. of participants implemented the activity)**	**Daily, times/day**	**Percentage of intervention days practiced, %** ^**1**^
Strength	Squatting (*n* = 9)	1.9 ± 1.0	64.5 ± 16.7%
	Lunging (*n* = 10)	1.6 ± 0.7	58.3 ± 21.2%
	Sit-to-stand (*n* = 6)	2.3 ± 0.9	63.0 ± 25.0%
	Toe standing and walking (*n* = 1)	1.0 ± 0	25.0 ± 0%
	Heel standing and walking (*n* = 1)	1.0 ± 0	75.0 ± 0%
	Stair climbing (*n* = 9)	2.2 ± 1.4	61.3 ± 20.2%
	Move sideways (*n* = 7)	1.5 ± 0.7	57.0 ± 20.1%
	Tightening muscles (*n* = 8)	1.6 ± 0.5	74.2 ± 22.9%
	Total value (*n* = 51)	1.6 ± 0.9	59.8 ± 21.1%
Neuromotor function	Tandem stand (*n* = 13)	1.7 ± 0.4	66.3 ± 20.0%
	One-leg stand (*n* = 15)	2.0 ± 1.0	68.2 ± 18.9%
	Tandem walk (*n* = 15)	1.8 ± 0.8	57.6 ± 20.2%
	Leaning (*n* = 8)	1.6 ± 0.6	67.1 ± 16.1%
	Stepping over objects (*n* = 3)	3.0 ± 1.1	68.8 ± 15.0%
	Stepping and changing direction (n = 0)	-	-
	Square stepping, hopping, or jumping (*n* = 8)	1.6 ± 0.8	46.1 ± 20.3%
	Total value (*n* = 61)	1.9 ± 0.8	62.4 ± 18.4%
Physical activity	Walk longer (*n* = 7)	1.9 ± 1.5	58.2 ± 26.4%
	Walk faster (*n* = 12)	2.3 ± 3.1	60.5 ± 21.6%
	Sit less (*n* = 5)	2.5 ± 2.2	59.4 ± 26.4%
	Break up sitting (*n* = 6)	2.0 ± 1.1	67.0 ± 17.3%
	Total value (*n* = 30)	2.2 ± 2.0	61.3 ± 23.0%

The data are presented as the mean ± SD or in %. The wLiFE55 + activities implemented during the intervention period are given. ^1^Percentage of days the activity was practiced in relation to the total days of the intervention. *n* = 15 were included in the analysis, but not all participants implemented each activity type, as indicated in the table

The weekly frequency of practice ranged between 25 and 75% days/week depending on the activity. For wLiFE55 + activities implemented by more than three participants, the highest frequencies were reported for *tightening muscles* (strength module), *one-leg stand* (neuromotor function module) and *break up sitting* (PA module), and the lowest frequencies were reported for *toe standing and walking, square stepping, leaning, hopping or jumping and walking longer*. (Table 3).

The daily frequency of practice ranged between 1 and 2.99 times per day depending on the activity. The highest frequencies were reported for *sit-to-stand (strength module), stepping over objects* (neuromotor function module), and *sit less* (PA module), and the lowest frequencies were reported for *toe/ heel standing and walking, square stepping, hopping or jumping, leaning and walking longer*. (Table 3).

### Muscle soreness

Four participants reported muscle soreness after beginning the intervention, but none reported worsening pain or prolonged exercise-related symptoms from the wLiFE55 + activities.

### *Acceptability of wLiFE55* + *activities*

Participants included in the analysis (*n* = 14) found it rather easy to integrate wLiFE55 + activities into their commutes. They perceived it more challenging to integrate them into daily work routines and non-work-related activities (Table 4). Most of the participants perceived some intervention-related improvements in neuromotor function, strength, and PA. Most felt safe when performing unsupervised wLiFE55 + activities and found it physically demanding. There was moderate or strong consensus for all items.

**Table 4:** Results of the acceptability of wLiFE55 + activities (*n* = 14^a^)

Category	Median (IQR)
**Integrability**^b^	
How easy or difficult did you find it to integrate the wLiFE55 + activities into your daily work routine?	3(1)
How easy or difficult have you found it to integrate the wLiFE55 + activities into your commutes to and from work?	4.5(1)
How easy or difficult did you find it to integrate the wLiFE55 + activities into your everyday life outside of work? (except work)	3.5(1)
**Perceived improvement of neuromotor function/strength/physical activity**^c^	
Do you feel that your neuromotor function has improved through the wLiFE55 + activities?	4(1)
Do you feel that your strength has improved through wLiFE55 + activities?	4(0)
Do you feel that your physical activity has increased as a result of the wLiFE55 + activities?	4(0)
**Satisfied with progress in neuromotor function/Strength/Physical activity**^d^	
Measured by your effort, how satisfied are you with your progress regarding your neuromotor function?	4(0.75)
Measured by your effort, how satisfied are you with your progress in terms of strength?	4(0)
Measured by your effort, how satisfied are you with your progress in terms of physical activity?	4(1)
**Perceived safety**^e^	
How safe did you feel in performing the wLiFE55 + activities during the sessions with the personal trainer?	6(0.75)
How safe did you feel doing the wLiFE55 + activities independently at home and in the office?	5(1)
**Physically demanding**^f^	
How physically demanding did you find the wLiFE55 + activities?	2.5(1)

The data are presented as the median and interquartile range (IQR) for each item. Post-Questionnaire with items regarding the acceptability of wLiFE55 + activities. ^a^: One accepatability questionnaire was not completed, and the value was missing. ^b^:1 = very difficult, 6 = very easy; ^c^:1 = not at all, 6 = very; ^d^:1 = very unsatisfied, 6 = very satisfied; ^e^:1 = very unsafe, 6 = very safe; ^f^:1 = very demanding, 6 = very easy

### *Acceptability of the wLiFE55* + *program*

Participants rated wLiFE55 + as good, highly recommended it to a friend or colleague and were willing to continue the program in the future (Table 5). Many participants found it ‘rather helpful’ to learn wLiFE55 + in a group with others. They were quite satisfied with their results from the wLiFE55 + program. Participants enjoyed the personal trainer sessions and found the instructions and exchanges very helpful. Repeated practice of wLiFE55 + activities and discussing specific activity situations with the personal trainer were rated as helpful. Theoretical content about the wLiFE55 + program was found to be useful, while the ‘if–then’ sentences for planning the wLiFE55 + activities were perceived only as ‘rather helpful ‘.

**Table 5:** Results of the acceptability of the wLiFE55 + program (*n* = 14^a^)

Category	Median (IQR)
**General aspects**	
What grade would you give the wLiFE55 + program?^b^	5(1)
Would you recommend the wLiFE55 + program to friends/colleagues?^c^	6(1)
Will you continue the program in the future?^c^	5(1)
How helpful would it have been to learn the wLiFE55 + program together in a group with other participants?^d^	3.5(2)
How satisfied are you with your results from the wLiFE55 + program?^c^	5(1)
**Personal trainer**	
Did you enjoy the session with the personal trainer?^c^	5.5(1)
Did you enjoy exercising on your own?^c^	5(0.75)
Were the instructions during the training sessions sufficient?^c^	6(0)
How helpful did you find the exchange with your personal trainer?^d^	6(0.75)
How helpful did you find the repetition of the wLiFE55 + activities during the session with the personal trainer?^d^	5(1)
How helpful did you find discussing wLIFE55 + activities situations with your personal trainer?^d^	5.5(1)
How helpful did you find it to do the respective wLiFE55 + activities directly in the specific everyday situation with your trainer during the sessions at your workplace?^d^	5(1)
**Content**	
How helpful did you find the teaching of theoretical content about the wLiFE55 + program?^d^	5(1)
How helpful did you find the 'if–then' sentences for planning the wLiFE55 + activities?^d^	4(2.75)
**Manual**	
How helpful did you find the manual (participant's handbook) for the wLiFE55 + program?^d^	5(0.75)
How helpful did you find the workbook for the wLiFE55 + program (ring binder with wLiFE55 + activities planners)?^d^	5(1.75)

The data are presented as the median and interquartile range (IQR). Post-Questionnaire with items regarding the acceptability of the wLiFE55 + program. ^a^:One questionnaire was not completed, and the value was missing. ^b^:1 = insufficient, 6 = very good; ^c^:1 = not at all, 6 = very; ^d^:1 = not at all helpful, 6 = very helpful

### Activity preferences

**Fig. 2:**
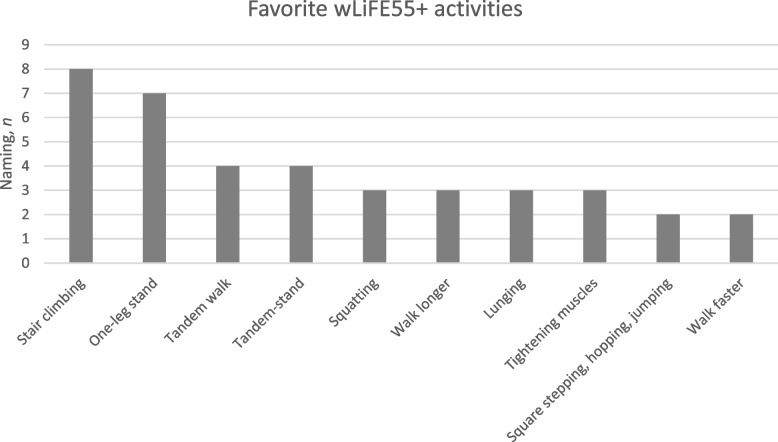
Favorite wLiFE55 + activities. The participants could list up to three wLiFE55 + activities (*n* = 15)

S*tair climbing* was the most frequently mentioned as the favorite activity, followed by *one-leg stand* and *tandem-walk*.* Square stepping*, *hopping*, *jumping* and *walking faster* were not among the favorite wLiFE55 + activities. (Fig. 2).

### Exploratory pre-post measures of subjective assessments

Balance confidence did not change from pre to post. The descriptive IPAQ results and small effect sizes (*r* = **-**0.162-0.385) indicated increased PA, although the difference was not significant (*p* = 0.255-0.616). (Table 6).

**Table 6:** Exploratory pre-post measures for subjective assessments for the total sample (*n* = 15)

Subjective Assessment	Pre Score	Post Score	Effect size	*p*-value
**ABC-D Scale**				
normal activity, Score	100 (1.6)	100 (2.3)	*r* = .179	*p* = .735
complex activity, Score	94 (11.7)	92.2 (8.1)	*r* = -.091	*p* = .824
**IPAQ Questionnaire**				
walking activity time, MET minutes/week	462 (445.5)	594 (1105.5)	*r* = -.385	*p* = .255
moderate activity time, MET minutes/week	1080 (1805)	1360 (1760)	*r* = -.162	*p* = .616
vigorous activity time, MET minutes/week	0 (540)	0 (580)	*r* = -.244	*p* = .552

The data are presented as the medians and interquartile range (IQR). *ABC-D Scale* Activities-Specific Balance Confidence Scale, *IPAQ* International Physical Activity Questionnaire

### Exploratory pre-post measures of objective assessments for the total sample

For neuromotor function, the descriptive 12-LBS and CBM results and medium effect sizes (12-LBS: *r* = -0.600, CBM: *r *= -0.667) indicated increased neuromotor performance, with significance for CBM (*p* = 0.030).

For strength, the descriptive 60CST results increased with a medium effect size (*r* = -0.462).

The results for activity-related PA, standing time, PA, and steps suggested increased activity-related PA with small effect sizes (r = -0.209-0.429). The results of the repetitions from sit-to-stand transfers and small effect size (*r* = -0.538) indicated increased sit-to-stand transfers per day.

For sedentary-related PA, the descriptive sedentary behavior results and medium effect size (*r* = 0.516) indicated reduced sedentary time. For sedentary bouts > 20 min, > 30 min and > 60 min, the descriptive sedentary time decreased from pre to post, with medium effect sizes (*r* = 0.560-0.736). The number of sedentary bouts > 20 min, > 30 min, and > 60 min decreased with small to medium effect sizes (*r* = -0.055-0.648), with a significant difference for sedentary bouts > 60 min (*p* = *0.0*40). (Table 7).

**Table 7:** Exploratory pre-post measures for objective assessments for the total sample

Objective Assessment	Pre Score	Post Score	Effect size	*p*-value
**Neuromotor function**				
12-LBS, Score (*n* = 15)	7 (1.5)	7 (1)	*r* = -.600	*p* = .095
CBM Scale, Score (*n* = 14)^a^	77.3 (14.9)	85.4 (10.9)	*r* = -.667	*p* = .030
**Strength (*n* = 14)**^a^				
60CST, number of repetitions	42 (17.8)	44 (8.5)	*r* = -.462	*p* = .169
**Physical activity (** ***n*** ** = 13)**^b^				
**Activity-related PA**				
Standing time, min	202.6 (66.2)	219.8 (127)	*r* = -.209	*p* = .542
Physical activity time, min	102.8 (22.8)	100.4 (36)	*r* = -.429	*p* = *.*191
Steps, number	8144 (2049.6)	8586.8 (3983.7)	*r* = -.363	*p* = *.*273
Sit-to-stand transfer, number	38.2 (11.3)	39.8 (6.5)	*r* = -.538	*p* = .094
**Sedentary-related PA**				
Sedentary time, min	647.4 (49.8)	613.4 (68.1)	*r* = .516	*p* = *.*110
Sedentary bouts > 20 min, min	485.8 (101.2)	434.3 (94.6)	*r* = .626	*p* = *.*048
Sedentary bouts > 30 min, min	387.0 (159.4)	336.0 (80.8)	*r* = .736	*p* = *.*017
Sedentary bouts > 60 min, min	219 (189.3)	148.6 (81.9)	*r* = .560	*p* = *.*080
Sedentary bouts > 20 min, number	10.4 (1.8)	9.7 (1.8)	*r* = -.055	*p* = *.*893
Sedentary bouts > 30 min, number	7.0 81.7)	6.3 (0.9)	*r* = .429	*p* = *.*191
Sedentary bouts > 60 min, number	2.8 (1.6)	1.7 (1.1)	*r* = .648	*p* = *.*040

The data are presented as the medians and interquartile range (IQR). *12-LBS* 12-Level Balance Scale, *CBM* Community Balance and Mobility Scale, *60CST* 60sec Chair Stand Test. ^a^: For the CBM and 60CST, one participant was not able to finish the post assessment due to pain with in the knees and hips. ^b^: For PA, one participant had problems wearing the electrode and experienced skin irritation, and one participant swam with the accelerometer. Both data sets were excluded from the analysis due to technical errors

### Exploratory pre-post measures of objective assessments for low-function participants

Subanalysis revealed a statistically significant difference in neuromotor function (12-LBS: *r* = -1.00; *p* = *0.0*26, CBM: *r* = 0.857; *p* = *0.0*47) and strength (60CST: *r *= 1.00; *p* = *0.0*36) between the low functioning participants in the pre-post comparison, with improvements from pretest to posttest. For PA, sit-to-stand transfer increased significantly (*p* = 0.016) with a large effect size (*r* = 1.000) (see Supplementary file 8).

## Discussion

This study indicates the feasibility of wLiFE55 + , as shown by measures of adherence and acceptance. Successful recruitment of the target population and overall positive feedback highlight the potential of wLiFE55 + as an innovative WPAI. For each wLiFE55 + domain (i.e. strength, neuromotor function, PA), several wLiFE55 + activities were integrated into commutes, during work, and daily life. Furthermore, our results suggest significant intervention-related pre-post increases in neuromotor function and PA parameters related to sedentary behavior. Participants with low baseline performance will benefit from wLiFE55 + .

### Adherence

High drop-out rates (22%) have been reported for WPAIs using exercise approaches conducted in addition to working hours [47]. With wLiFE55 + , the drop-out rate is much lower (12%), less time is required for training, and integration into everyday activities is easier.

Intervention adherence (100%) was greater than that for other WPAIs, for which adherence rates ranging from 57–99% have been reported [48]. We realized that the participants appreciated the personal trainer sessions, which is consistent with other studies [49]. These intervention sessions not only motivated our participants, but also provided flexibility by allowing rescheduling to avoid conflicts with other appointments. The results suggest that one-on-one personal training is an effective method for changing attitudes and increasing the amount of PA [50].

The average activity adherence was 58% with 9 wLiFE55 + activities out of 16 implemented. Participants could decide on the number of wLiFE55 + activities according to their time capacity. The mean activity adherence for the three domains (3.7–2.2 wLiFE55 + activities/per week) in our study was lower than that in previous LiFE studies summarized in reviews (4.9 wLiFE55 + activities/per week) [22, 25, 51]. In contrast to all previous LiFE studies, our target population was younger, not retired, and relatively healthy. Therefore, our participants’ subjective need to integrate wLiFE55 + activities may have been lower than that reported in previous studies in older, more impaired target populations.

### Frequency of practice

A higher frequency of practice is generally associated with greater effects [52]. Unlike structured training programs using multiple sets and rest intervals for counting the frequency of practice [53], wLiFE55 + integrates activities into daily routines, resulting in repetitions throughout the week.

Similar to aLiFE, neuromotor activities were practiced most frequently by the majority of participants, followed by strength activities and PA. Participants may have prioritized neuromotor activities due to perceived deficits, while their relatively high baseline PA status may have reduced interest in further increasing activity levels. According to previous studies, time constraints and employment status may explain the lower practice frequency among retired participants [22]. Nevertheless, our exploratory analysis suggested that even with reduced implementation, wLiFE55 + positively impacts physical capacity in our employed population.

### Muscle soreness

Challenging exercises are associated with numerous positive effects [54]. Only a few participants reported muscle soreness after starting the intervention. This disappeared during the study, suggesting that wLiFE55 + activities did not overtax the participants. Muscle soreness is a common training effect for untrained individuals [55, 56].

### Acceptability of the wLiFE55 + activities

The integrability of wLiFE55 + activities varied with the environment. Commuting had the highest integrability, likely due to lower stress, less observation, and ample space for wLiFE55 + activities. However, integrability was lower in the workplace and daily life, possibly due to time constraints and cognitive demands [57]. The acceptance of wLiFE55 + activities differed by domain. Participants perceived the most improvement in strength and PA, whereas assessing neuromotor function was less popular. Whenever participants executed wLiFE55 + activities they felt safe and rated the wLiFE55 + activities as physically demanding. This justifies further investments in the development of wLiFE55 + .

Previous studies have shown that an employee’s setup at work affects their movements at work, such as walks to the coffee machine [58]. Therefore, differences in workplaces may also affect the integration of the wLiFE55 + activities. Such influence may be measured in future studies by systematically analyzing the workplace configuration [59].

### Acceptability of the wLiFE55 + program

Participants showed good acceptance of the wLiFE55 + program. A "good" rating indicates successful intervention development, and opinions on learning the program in a group varied, possibly due to individual preferences. Personalized approaches offering both individual- and group-based LiFE training may enhance acceptance [60–62]. The high enjoyment levels during personal trainer sessions and nonsupervised LiFE training highlight the intervention's potential for older employees.

The results show that the use of personal trainers sessions is very important for the high acceptability and adherence of the wLiFE55 + program. On the same note, personal trainer sessions may also pose some challenges for its practical implementation including costs and lost working time for the company (if the personal training sessions take place during working hours). In a previous LiFE study, some personal trainer sessions were replaced by telephone calls after a few weeks [63], which may reduce the costs. The use of a group program with one personal trainer session for several participants may also be an option for more cost-effective implementation [63].

### Activity preferences

Three of the most favorably rated exercises are from the neuromotor function module, indicating a greater acceptance of neuromotor training among older employees. These wLiFE55 + activities can be performed in limited space without causing sweating. The participants found the neuromotor exercises to be novel, and their motivation to improve may have been high due to the challenges shown in the pretest. In the strength module, *stair climbing* was the preferred activity, likely because it can be easily incorporated into daily life.

### Exploratory pre-post measures of subjective assessments

The limited effects observed for balance confidence are related to ceiling effects, as shown by the high baseline values.

### Exploratory pre-post measures of objective assessments for the total sample

The large effect size and significant improvement observed in CBM performance may suggest a clinically relevant effect on balance and mobility performance for older employees. These findings are consistent with previous LiFE studies [24, 25] Our participants already had a very good balance and mobility, as indicated by a CBM score of 81.7 points (normal value 50–59 years: 77 points; 60–69 years: 65 points) [44]. Nevertheless, our findings suggest that the wLiFE55 + program could further improve balance for these participants.

The medium effect (*r* = 0.462) observed in the 60CST may suggest a training effect induced by wLiFE55 + . This result is in line with short-term strength exercises of 4 weeks [64], and greater improvement in strength performance can be expected for long-term interventions (12 weeks) [65].

For activity-related PA, each variable increased, leading to the assumption that PA improved with increasing wLiFE55 + activities. The participants showed, on average, a 10 min longer standing and PA time for the posttest. The number of steps increased, including the wLiFE55 + activities for PA.

For sedentary-related PA, each variable decreased. The total sedentary time, as well as the sedentary bouts > 20 min, > 30 min, and > 60 min, decreased significantly which is consistent with the activity preferences for *break-up sitting* and *sitting less* [66].

Based on our proof-of-concept study, the majority of participants seem to benefit from wLiFE55 + . On the same note, few participants may not benefit from the intervention for specific outcomes such as strength or sedentary behavior. A responder analysis needs to be conducted in a larger study.

### Exploratory pre-post measures of objective assessments: low function participants

The analyzed dose–response relationship between baseline performance and improvement through the wLiFE55 + program showed that low function participants improved significantly in neuromotor function (12-LBS and CBM), strength, and PA for sit-to-stand transfers. Compared to the total sample, the effects were greater in all domains, and the improvement became significant in the 12-LBS, 60CST and sit-to-stand transfer. Participants with low function, in particular, benefited from the wLiFE55 + activities, which is consistent with previous literature [67]. Further analysis is necessary to determine the optimal doses for improving physical capacity and activity [68].

### Refinement of wLiFE55+ 

Based on the pilot study, we propose four major refinement suggestions for items with two points below the maximum score in the acceptance questionnaire. First, the wLiFE55 + program will be shortened for wLiFE55 + activities with the lowest frequency. *Toe walking* and *heel walking* for the strength module, and *stepping and changing direction* for the neuromotor function module will be excluded. For the PA module, no wLiFE55 + activity was excluded, as it consisted of only four wLiFE55 + activities. Second, for the category integrability, we wanted to improve the integration of wLiFE55 + activities into daily work routines. Therefore, we will use qualitative data to better understand the difficulties of the participants (published separately). Third, for the two categories of perceived improvement and satisfaction with progress for the three domains, we propose increasing the difficulty of the wLiFE55 + activities and increasing the duration of the intervention. Fourth, for helpfulness learning wLiFE55 + in a group, we suggest developing a mixed design of group sessions and individual personal trainer sessions consistent with previous results [69].

### Strategies to maintain motivation

One option for future wLiFE55 + studies is the integration of a digital approach that allows participants to track their progress and record their goals. The feasibility of such an approach for implementing aLiFE in retired seniors has been previously demonstrated [70] and might be translatable to wLiFE55 + . Digital technology may foster visualization of the participant’s current level of performance, setting goals, and allow objective assessment of their progress by activity tracking. A smartphone app may allow participants to document their wLiFE55 + activities and progress via a special app. Studies have shown that visualizing progress, for example, through progress bars, can increase participants' motivation and promote health-promoting behavioral changes [71–73]. An app may also foster participant-trainer interaction. Personalized attention allows us to address individual needs and progress and support participants during the intervention [19, 69, 74].

Another strategy for boosting motivation could be gamification. In this context, there is the possibility of introducing elements such as point systems or leaderboards to further motivate participants and encourage long-term participation. These approaches have already been shown to be effective, especially in older adults [75, 76].

Collaborations with health insurance companies and their bonus programs may offer participants a financial incentive for long-term participation in wLiFE55 + . In summary, several options exist to further develop wLiFE55 + to foster participant motivation and achieve sustainable implementation of our training program.

### Adaptation

Future studies could adapt wLiFE55 + to different work environments such as manufacturing. Upper body exercises could be established, as work in production sometimes requires good upper limb strength [77]. In general, adaptations of the wLiFE55 + to other jobs should be based on a structured framework to determine the exact needs of workers in their workplace [31].

### Limitations

Our study used a convenient sample of university employees who possess a high education level and flexibility in organizing work. The findings may not apply to older employees in different occupations and working conditions [78]. The convenience sample including a small number of participants limits the generalizability of our findings. To overcome these limitations, the next step involves consecutively recruiting larger samples of older employees from a broader range of working contexts to investigate the effects of wLiFE55 + . The feasibility of personal trainer sessions in real life scenarios (e.g., companies) needs to be proven.

However, the present sample was sufficient for testing the initial feasibility of the wLiFE55 + program. Although the short intervention period allowed us to assess the acceptability of wLiFE55 + and identify areas for further development, longer interventions are necessary to evaluate behavior change and adoption of the program [31]. The implementation of numerous wLiFE55 + activities within a short timeframe may have overwhelmed some participants, but it also helped us identify the most accepted wLiFE55 + activities within the target population. Longer intervention periods would allow participants to establish habits of their preferred activities and progressively increase the difficulty level for optimal task challenges. As we lacked a control group, we cannot draw definitive conclusions about the effectiveness of wLiFE55 + . For the pilot study we deliberately did not use a control group due to the exploratory, hypothesis-generating nature of the study. The aim was not to prove the effects of wLiFE55 + . Additionally, the subanalysis low functioning sample was small. Nonetheless, our findings suggest that individuals with lower functional performance may benefit more and could be the focus of future studies.

### Impact and application for future research

Our study shows the successful development of a new innovative WPAI specifically designed for counteracting the functional decline of older employees by carrying out strength and neuromotor exercises. To the best of our knowledge, it is the first WPAI program integrating exercise into daily routines at the workplace. The high level of adherence and acceptance supports the success of the wLiFE55 + intervention development.

As we gave the participants a choice of exercises, we were able to identify the most suitable exercises (e.g. climbing stairs, one-leg stand, tandem walk) building the basis for future studies. Our findings highlight the importance of a personal trainer in the context of program acceptance. This finding should be incorporated into the further development of wLiFE55 + . To reduce the costs of a personal trainer, the wLiFE55 + program may be examined in a group format, previously developed for retired adults [63]. Using such a format, older employees could exchange ideas on the best ways to integrate training in the workplace.

Based on our pilot study, the next step is evaluating the effectiveness of wLiFE55 + within a follow-up study including a longer intervention period and a consecutively recruited sample. We would like to address the following questions in the next study: Can the wLiFE55 + program significantly improve neuromotor function, strength and PA in comparison to other WPAIs such as exercise courses? Can long-term effects be achieved? Can the wLiFE55 + program be transferred to other working environments? Can the wLiFE55 + program reduce the number of days employees are absent from work? The transfer of wLiFE55 + to people with a low level of education is another future field of research.

## Conclusion

This pilot study demonstrated the feasibility of using wLiFE55 + to improve health-related factors in older adults, including neuromotor function, strength, and PA. The development stages of wLiFE55 + were successful and involved community access, expert consultations, and the creation of a participant manual. The pilot study showed significant improvements in these factors after a 4-week intervention. The positive feedback from participants justifies the continuation of wLiFE55 + as a supplementary WPAI for older employees. This study addresses previous shortcomings in integrated WPAIs by targeting the needs of older employees and achieving high adherence. This study represents an important step toward preventing functional decline and serves as a basis for future evaluation and implementation of wLiFE55 + .

### Supplementary Information

Supplementary Material 1: Supplementary File 1. Short description of the participant manual. The participant manual is currently available in German and serves as a guide for participants during the program. It is handed out to participants at the first training session. It explains the scope of the wLiFE55+ program and offers strategies for seamless integration into everyday working life. The first section of the manual focuses on the key aspects of the program, particularly the concepts of habit formation and activity integration. As the wLiFE55+ program aims for long-term implementation, the importance of setting personal goals is also clear. In the following chapters "Neuromotor Function","Strength" and "Physical Activity", the importance of these components for the health of the target group is discussed, and the effect of targeted activities is illustrated. The principles of wLiFE55+ are presented and the individual activities (neuromotor function: e.g., tandem stand, strength: e.g., squat, physical activity: e.g., interrupting sitting phases) are described and illustrated in detail. The participant manual is designed to help participants organize their training independently. Each chapter therefore offers strategies for recognizing situations, selecting suitable activities, and adjusting the intensity accordingly. For example, the chapter on balance shows how participants can optimize their balance training by making small adjustments (e.g., reducing the support surface). Supplementary File 2. Checklist personal trainer session for teaching the wLiFE55+ program to the study participants during 4 personal trainer sessions at the workplace. Supplementary File 3. Daily Routine chart. Supplementary File 4. wLiFE55+ assessment tool (version March 2022; adapted version of the aLAT [24]). Supplementary File 5. Activity Planner. Supplementary File 6. The extension process from the Short Physical Performance Battery (SPPB) to the 8-Level-Balance-Scale (8-LBS) to the 12-Level-Balance-Scale (12-LBS) to assess static balance for older employees. The SPPB includes three balance performance items (Romberg stand, semi-tandem stand, tandem stand; each with eyes open for 10 sec) with a maximum score of 4 points. The 8-LBS is an extension of the SPPB [23]. The 8-LBS performed in previous standing positions (Romberg stand, semi-tandem stand and tandem stand) for a longer time (30 sec instead of 10 sec) and with two different conditions (eyes open, eyes closed). In addition, a fourth standing position (one-leg stand on the preferred leg) is used. This is performed with eyes open, eyes closed, and eyes closed with an additional cognitive distractor. The 8-LBS has a maximum score of 8 points. The 12-LBS is an extension of the 8-LBS. The one-leg stand on the nonpreferred leg was added and performed with all three conditions (eyes open, eyes closed, eyes closed with an additional cognitive distractor). The 12-LBS has a maximum score of 12 points. This extension process was performed to avoid ceiling effects, as older employees show a better static balance than do retired seniors. Supplementary File 7. Descriptive data for working situation items (*n*=17).*Notes. *Self-developed questionnaire with items regarding working situation-related data. The questionnaire data for two participants were missing. Supplementary File 8. Exploratory pre-post measures for objective assessment of low function participants. Notes. The data are presented as the medians and interquartile range (IQR). 12-LBS= 12-Level Balance Scale; CBM=Community balance and mobility scale; 60CST= 60sec Chair Stand Test. Supplementary File 9. Exploratory pre-post measures for objective assessment

## Data Availability

All data generated or analyzed during this study are included in this published article and its supplementary information files.

## Abbreviations

PA: Physical Activity
WPAI: Workplace Physical Activity Intervention
LiFE: Lifestyle-integrated Functional Exercise
aLiFE: adapted Lifestyle-integrated Functional Exercise for young retired seniors
wLiFE55 +: workLiFE55 +
ACSM: American College of Sports Medicine
wLAT55 +: wLiFE55 + Assessment Tool
HAPA: Health Action Process Approach
ABC-D: Activities-Specific Balance Confidence-Scale
IPAQ: International Physical Activity Questionnaire
12-LBS: 12-Level Balance Scale
8-LBS: 8-Level Balance Scale
CBM: Community Balance and Mobility Scale
60CST: 60sec Chair Stand Test
IQR: Interquartile Range
BMI: Body Mass Index
