# Supplementary material for: The work Lifestyle-integrated Functional Exercise program for preventing functional decline in employees aged 55 years and older: development and initial evaluation
Source: Eur Rev Aging Phys Act. 2024 Aug 6;21:21. doi: 10.1186/s11556-024-00356-5 (PMC11304822; doi:10.1186/s11556-024-00356-5)
Supplement: Supplementary file 1 — Supplementary Material 1: Supplementary File 1. Short description of the participant manual. The participant manual is currently available in German and serves as a guide for participants during the program. It is handed out to participants at the first training session. It explains the scope of the wLiFE55+ program and offers strategies for seamless integration into everyday working life. The first section of the manual focuses on the key aspects of the program, particularly the concepts of habit formation and activity integration. As the wLiFE55+ program aims for long-term implementation, the importance of setting personal goals is also clear. In the following chapters "Neuromotor Function","Strength" and "Physical Activity", the importance of these components for the health of the target group is discussed, and the effect of targeted activities is illustrated. The principles of wLiFE55+ are presented and the individual activities (neuromotor function: e.g., tandem stand, strength: e.g., squat, physical activity: e.g., interrupting sitting phases) are described and illustrated in detail. The participant manual is designed to help participants organize their training independently. Each chapter therefore offers strategies for recognizing situations, selecting suitable activities, and adjusting the intensity accordingly. For example, the chapter on balance shows how participants can optimize their balance training by making small adjustments (e.g., reducing the support surface). Supplementary File 2. Checklist personal trainer session for teaching the wLiFE55+ program to the study participants during 4 personal trainer sessions at the workplace. Supplementary File 3. Daily Routine chart. Supplementary File 4. wLiFE55+ assessment tool (version March 2022; adapted version of the aLAT [24]). Supplementary File 5. Activity Planner. Supplementary File 6. The extension process from the Short Physical Performance Battery (SPPB) to the 8-Level-Balance-Scale (8-LBS) to the 1 [file 11556_2024_356_MOESM1_ESM.docx]

**Supplementary File 1: Short description of the participant manual**

The participant manual is currently available in German and serves as a guide for participants during the program. It is handed out to participants at the first training session. It explains the scope of the wLiFE55+ program and offers strategies for seamless integration into everyday working life.

The first section of the manual focuses on the key aspects of the program, particularly the concepts of habit formation and activity integration. As the wLiFE55+ program aims for long-term implementation, the importance of setting personal goals is also clear.

In the following chapters "Neuromotor Function", "Strength" and "Physical Activity", the importance of these components for the health of the target group is discussed, and the effect of targeted activities is illustrated. The principles of wLiFE55+ are presented and the individual activities (neuromotor function: e.g., tandem stand, strength: e.g., squat, physical activity: e.g., interrupting sitting phases) are described and illustrated in detail.

The participant manual is designed to help participants organize their training independently. Each chapter therefore offers strategies for recognizing situations, selecting suitable activities, and adjusting the intensity accordingly. For example, the chapter on balance shows how participants can optimize their balance training by making small adjustments (e.g., reducing the support surface).

**Supplementary File 2: Checklist personal trainer session for teaching the wLiFE55+ program to the study participants during 4 personal trainer sessions at the workplace**

| **Session, week number** | **What the wLiFE55+ trainer will do** | **Time** |
| --- | --- | --- |
| Session 1: workplace visit  week 1 | - Introduce the wLiFE55+ trainer and program by use of the wLiFE55+ manual - Evaluate the ability and opportunities for wLiFE55+ activities using the wLiFE55+ Assessment Tool (wLAT55+) - Talk about Goal setting about short- and longterm physical goals - Implement up to four wLiFE55+ activities (neuromotor function, strength, PA) linked to specific daily tasks, situations or places using the Daily Routine Chart and the Activity Planner | 1.5 hour |
| Session 2: workplace visit,  week 2 | Continue teaching and implementing the wLiFE55+ program   - Clarify any questions related to the wLiFE55+ program - Review the activities commenced previously, Activity Planner - Teach ways of making the program more effective (upgrade activities by increasing the task challenge as shown in the wLAT55+) - Implement up to four new (neuromotor function, strength, and physical activity) exercises. Link these activities to specific working tasks, situations or places. - Develop plans for embedding the activities - Increase the autonomy of participants in selecting opportunities to embed activities in daily tasks and in upgrading the exercise tasks. - Use the Activity Planner to record plans and upgrades | 1.5 hour |
| Session 3: workplace visit,  week 3 | See workplace visit 2   - Clarify any questions related to the wLiFE55+ program - Implement up to four new (neuromotor function, strength, and physical activity) exercises - Continue to teach the wLiFE55+ program and upgrade existing exercises, increase autonomy of participants in planning activities and where can be embedded in daily tasks and routines | 1.5 hour |
| Session 4: workplace visit,  week 4 | See workplace visit 3   - Clarify any questions related to the wLiFE55+ program - Implement up to four new (neuromotor function, strength, and physical activity) exercises - Continue to teach the wLiFE55+ program and upgrade existing exercises, increase autonomy of participants in planning activities and where can be embedded in daily tasks and routines | 1.5 hour |

**Supplementary File 3. Daily Routine chart**

|  | **Monday** | **Tuesday** | **Wednesday** | **Thursday** | **Friday** | **Saturday** | **Sunday** |
| --- | --- | --- | --- | --- | --- | --- | --- |
|  | **Get up** | **Get up** | **Get up** | **Get up** | **Get up** | **Get up** | **Get up** |
| **Morning** |  |  |  |  |  |  |  |
|  | **On the way to work** | **On the way to work** | **On the way to work** | **On the way to work** | **On the way to work** | **On the way to work** | **On the way to work** |
|  |  |  |  |  |  |  |  |
|  | **In the office** | **In the office** | **In the office** | **In the office** | **In the office** | **In the office** | **In the office** |
|  |  |  |  |  |  |  |  |
|  | **Break** | **Break** | **Break** | **Break** | **Break** | **Break** | **Break** |
| **Afternoon** |  |  |  |  |  |  |  |
|  | **In the office** | **In the office** | **In the office** | **In the office** | **In the office** | **In the office** | **In the office** |
|  |  |  |  |  |  |  |  |
|  | **On the way at home** | **On the way at home** | **On the way at home** | **On the way at home** | **On the way at home** | **On the way at home** | **On the way at home** |
| **Evening** |  |  |  |  |  |  |  |
|  | **Dinner** | **Dinner** | **Dinner** | **Dinner** | **Dinner** | **Dinner** | **Dinner** |
|  |  |  |  |  |  |  |  |

**Supplementary File 4. wLiFE55+ assessment tool (version March 2022; adapted version of the aLAT [**[**24**](#_ENREF_24)**])**

| **Decreased base of support** | **Instructions** | **Level 1** | **Level 2** | **Level 3** | **Level 4** |
| --- | --- | --- | --- | --- | --- |
|  |  | ^(completed/not completed)^  🞏 🞏  yes no | ^(completed/not completed)^  🞏 🞏  yes no | 🞏 🞏 ^(completed/not completed)^  yes no | 🞏 🞏 ^(completed/not completed)^  yes no |
| Tandem stand | - Heel-to-toe stand - Hold for 15 sec | - Hold support   🞏 🞏  yes no | - No support   🞏 🞏  yes no | - With **one** additional challenge:   🞏 🞏 Manual: write an email  🞏 🞏 Mental  🞏 🞏 Eyes closed  🞏 🞏 Head turn  🞏 🞏 Pivot turn (without reactive step) | - With **two** additional challenges:   🞏 🞏 Manual + mental  🞏 🞏 Manual + eyes closed  🞏 🞏 Eyes closed + mental  🞏 🞏 Head turn + eyes closed  🞏 🞏 Pivot turn + eyes closed  🞏 🞏 ________ + ________ |
| Tandem walk | - Heel to toe walk - Walking distance 2.0 metres | - Hold support   🞏 🞏  yes no | - No support   🞏 🞏  yes no | - With **one** additional challenge:   🞏 🞏 Manual: carry glass of water  🞏 🞏 Mental  🞏 🞏 Eyes closed  🞏 🞏 Head turn  🞏 🞏 Crouching: pick item from floor | - With **two** additional challenges:   🞏 🞏 Manual + mental  🞏 🞏 Manual + eyes closed  🞏 🞏 Eyes closed + mental  🞏 🞏 Head turn + eyes closed  🞏 🞏 ________ + ________ |
| One-leg stand | - Support available - Hold for 15s sec - **Record least stable leg:**   🞏 Left 🞏 Right | - Hold support   🞏 🞏  yes no | - No support   🞏 🞏  yes no | - With **one** additional challenge:   🞏 🞏 Manual: brushing hair (simulate)  🞏 🞏 Mental  🞏 🞏 Eyes closed  🞏 🞏 Head turn  🞏 🞏 Standing scale: pick item from chair | - With **two** additional challenges:   🞏 🞏 Manual + mental  🞏 🞏 Manual + eyes closed  🞏 🞏 Eyes closed + mental  🞏 🞏 Head turn + Eyes closed  🞏 🞏 ________ + ________ |
| **Shifting weight to limits of stability** | **Instructions** | **Level 1**  ^(completed/not completed)^ | **Level 2**  ^(completed/not completed)^ | **Level 3**  ^(completed/not completed)^ | **Level 4**  ^(completed/not completed)^ |
| Leaning | - Lean as far as possible forward - Bend at ankle joint (not waist) - Hold for 10 seconds | - Feet shoulder-width apart - No support   🞏 🞏   - yes no | - Feet together - No support   🞏 🞏   - yes no | - Feet together - With **one** additional challenge:   🞏 🞏 Manual: write a note)  🞏 🞏 Mental  🞏 🞏 Eyes closed   - 🞏 🞏 One leg stand | - Feet together - With **two** additional challenges:   🞏 🞏 Manual + mental  🞏 🞏 Manual + eyes closed  🞏 🞏 Eyes closed + mental  🞏 🞏 One leg stand + manual  🞏 🞏 One leg stand + eyes closed   - 🞏 🞏 ________ + ________ |
| **Stepping over objects** | **Instructions** | **Level 1** | **Level 2** | **Level 3** | **Level 4** |
| Forward and backward | - Place foam block on floor - step forward and backward over block (30 cm high) | - Hold support   🞏 🞏   - yes no | - No support   🞏 🞏   - yes no | - With **one** additional challenge:   🞏 🞏 Manual: carrying a water bottle  🞏 🞏 Mental  🞏 🞏 Forward hopping over object (instead of stepping) | - With **two** additional challenges:   🞏 🞏 Manual task + mental  🞏 🞏 Hopping + mental   - 🞏 🞏 ________ + ________ |
| **Stepping, hopping, jumping in different ways (agility)** | **Instructions** | **Level 1** | **Level 2** | **Level 3** | **Level 4** |
| Stepping and changing direction | - Perform forward, backward, sideward stepping | - Step pattern “Level 1” - Manual page 44   🞏 🞏   - yes no | - Step pattern “Level 2” - Manual page 45   🞏 🞏   - yes no | - Step pattern “Level 3”   Manual page 45  🞏 🞏   - yes no | - **-** |
| Square stepping and hopping | - Perfom square stepping/ hopping - Document if stepping and/or hopping was performed | - Step pattern “Level 1” - Manual page 48   🞏 🞏  yes no  Stepping  🞏 🞏  yes no   - Hopping | - Step pattern “Level 2” - Manual page 48   🞏 🞏  yes no  Stepping  🞏 🞏  yes no   - Hopping | - Step pattern “Level 3” - Manual page 48   🞏 🞏 Stepping (completed/not completed)  yes no  🞏 🞏 Hopping (completed/not completed)  yes no | - **-** |
| Square stepping and hopping | - Perfom square stepping/ hopping - Document if stepping and/or hopping was performed | - Step pattern “Level 1” - Manual page 48   🞏 🞏  yes no  Stepping  🞏 🞏  yes no  Hopping | - Step pattern “Level 2” - Manual page 48   🞏 🞏  yes no  Stepping  🞏 🞏  yes no  Hopping | - Step pattern “Level 3” - Manual page 48   🞏 🞏 Stepping (completed/not completed)  yes no  🞏 🞏 Hopping (completed/not completed)  yes no | **-** |
| Square jumping | - Perfom square jumping | - Pattern “Level 1” - Manual page 49   🞏 🞏   - yes no | - Pattern “Level 2” - Manual page 49   🞏 🞏   - yes no | - Pattern “Level 3” - Manual page 49   🞏 🞏   - yes no | **-** |
| **Bend your knees** | **Instructions** | **Level 1** | **Level 2** | **Level 3** | **Level 4** |
| Squatting | - Have support available - The exercise must be pain free | - Quarter squat with no support - Hold for 5 seconds   🞏 🞏   - yes no | - Half squat with no support - Hold for 5 seconds   🞏 🞏   - yes no | - Quarter **one legged** squat with no support - Hold for 5 seconds   🞏 🞏   - yes no | - Half **one legged** squat with no support - Hold for 5 seconds   🞏 🞏  yes no |
| Lunging | - Have support available - The exercise must be pain free | - Partial lunge - During standing - Hold for 5 seconds   🞏 🞏   - yes no | - Partial lunge - During walking (4m)   🞏 🞏   - yes no | - Full lunge - During standing - Hold for 5 seconds   🞏 🞏   - yes no | - Full lunge - During walking (4m)   🞏 🞏   - yes no |
| **Sit to stand** | **Instructions** | **Level 1** | **Level 2** | **Level 3** | **Level 4** |
| Standing up from a seated position | - Sit with bottom to front of chair - Push up from legs. | - Rising from a chair - no hand support   🞏 🞏   - yes no | - Rising from a chair slowly - No hand support - Must take at least 5 seconds   🞏 🞏   - yes no | - Rising from a standard chair - Approx 75% body weight on left/right leg (on your toe) - No hand support   🞏 🞏   - yes no | - Rising from a standard chair slowly - Approx 75% body weight on left/right leg (on your toe) - No hand support - Must take at least 5 seconds   🞏 🞏   - yes no |
| **On your toes** | **Instructions** | **Level 1** | **Level 2** | **Level 3** | **Level 4** |
| Walking on toes | - Heels must be off the ground | - Hold support - Distance 2 m   🞏 🞏   - yes no | - No support - Heels high - Distance 4 m   🞏 🞏   - yes no | - No support - Heels high - Distance 8m   🞏 🞏   - yes no | - No support - Distance 16m - Heels high - Carrying a heavy item (e.g., working bag, water bottle crate)   🞏 🞏   - yes no |
| **On your heels** | **Instructions** | **Level 1** | **Level 2** | **Level 3** | **Level 4** |
| Walking on heels | - Toes must be off the ground | - Hold support - Distance 2 m   🞏 🞏   - yes no | - No support - Distance 4 m   🞏 🞏   - yes no | - No support - Distance 8m   🞏 🞏   - yes no | - No support - Distance 16m   🞏 🞏   - yes no |
| **Up the stairs** | **Instructions** | **Level 1** | **Level 2** | **Level 3** | **Level 4** |
| Stair climing | - Walk up the stairs - Use your legs rather than pulling up with your hands | - Walking up the stairs - No support   🞏 🞏   - yes no | - Walking up the stairs - Carrying a heavy item (e.g., laundry basket, water bottle crate   🞏 🞏   - yes no | - Walking up two stairs at a time - With support   🞏 🞏   - yes no | - Walking up two stairs at a time - Without support   🞏 🞏   - yes no |

**Supplementary File 5. Activity Planner**

| **BALANCE activities** | **Example of daily tasks. How, when and where?** | **Tick if done** | | | | | | |
| --- | --- | --- | --- | --- | --- | --- | --- | --- |
|  |  |  |  |  |  |  |  |  |
|  |  |  |  |  |  |  |  |  |
| *Tandem stand* |  |  |  |  |  |  |  |  |
| *Tandem walk* |  |  |  |  |  |  |  |  |
| *One-leg stand* |  |  |  |  |  |  |  |  |
| *Leaning forwards, backwards, sidewards* |  |  |  |  |  |  |  |  |
| *Stepping over objects* |  |  |  |  |  |  |  |  |
| *Stepping and changing direction* |  |  |  |  |  |  |  |  |
| *Square stepping and hopping* |  |  |  |  |  |  |  |  |
| *Square jumping* |  |  |  |  |  |  |  |  |

| **STRENGTH principle** | **STRENGTH activities** | **Example of daily tasks. How, when and where?** | **Tick if done** | | | | | | |
| --- | --- | --- | --- | --- | --- | --- | --- | --- | --- |
|  |  |  |  |  |  |  |  |  |  |
| **Bend your knees** | *Squatting* |  |  |  |  |  |  |  |  |
|  | *Lunging* |  |  |  |  |  |  |  |  |
| **Sit to stand** | *Normal chair* |  |  |  |  |  |  |  |  |
|  | *Low chair* |  |  |  |  |  |  |  |  |
|  | *One-legged* |  |  |  |  |  |  |  |  |
| **On your toes** | *Standing* |  |  |  |  |  |  |  |  |
|  | *Walking* |  |  |  |  |  |  |  |  |
| **On your heels** | *Standing* |  |  |  |  |  |  |  |  |
|  | *Walking* |  |  |  |  |  |  |  |  |
| **Up the stairs** | *One stair* |  |  |  |  |  |  |  |  |
|  | *Two steps at a time* |  |  |  |  |  |  |  |  |
| **Move legs sideways** | *Lying* |  |  |  |  |  |  |  |  |
|  | *Standing* |  |  |  |  |  |  |  |  |
|  | *Walking* |  |  |  |  |  |  |  |  |
| **Tighten muscles** | *Lying* |  |  |  |  |  |  |  |  |
|  | *Sitting* |  |  |  |  |  |  |  |  |

| **PHYSICAL**  **ACTIVITY principle** | **PHYSICAL**  **activities** | **Example of daily tasks. How, when and where?** | **Tick if done** | | | | | | |
| --- | --- | --- | --- | --- | --- | --- | --- | --- | --- |
|  |  |  |  |  |  |  |  |  |  |
| **Move more** | *Walk longer* |  |  |  |  |  |  |  |  |
|  | *Walk faster* |  |  |  |  |  |  |  |  |
| **Reducing sedentariness** | *Sit less* |  |  |  |  |  |  |  |  |
|  | *Break up sitting* |  |  |  |  |  |  |  |  |

**Supplementary File 6. The extension process from the Short Physical Performance Battery (SPPB) to the 8-Level-Balance-Scale (8-LBS) to the 12-Level-Balance-Scale (12-LBS) to assess static balance for older employees**

|  |  | **Short Physical Performance Battery (SPPB) [**[**80**](#_ENREF_80)**]** | **8-Level-Balance-Scale (8-LBS) [**[**23**](#_ENREF_23)**]** | **12-Level-Balance-Scale (12-LBS)** |
| --- | --- | --- | --- | --- |
| Romberg stand | eyes open | ≥ 10s (1 point) | ≥ 30s (1 point) | ≥ 30s (1 point) |
|  | eyes closed |  | ≥ 30s 1 point) | ≥ 30s (1 point) |
| Semi-tandem stand | eyes open | ≥ 10s (1 point) | ≥ 30s 1 point) | ≥ 30s (1 point) |
|  | eyes closed |  |  | ≥ 30s (1 point) |
| Tandem stand | eyes open | ≥ 10s (2 point) | ≥ 30s 1 point) | ≥ 30s (1 point) |
|  | eyes closed |  | ≥ 30s 1 point) | ≥ 30s (1 point) |
| One-leg- stand on preferred foot | eyes open |  | ≥ 30s 1 point) | ≥ 30s (1 point) |
|  | eyes closed |  | ≥ 30s 1 point) | ≥ 30s (1 point) |
|  | eyes closed with cognitive distractor |  | ≥ 30s 1 point) | ≥ 30s (1 point) |
| One-leg- stand on non-preferred foot | eyes open |  |  | ≥ 30s (1 point) |
|  | eyes closed |  |  | ≥ 30s (1 point) |
|  | eyes closed with cognitive distractor |  |  | ≥ 30s (1 point) |
| maximum score |  | 4 points | 8 points | 12 points |

The SPPB includes three balance performance items (Romberg stand, semi-tandem stand, tandem stand; each with eyes open for 10 sec) with a maximum score of 4 points.

The 8-LBS is an extension of the SPPB [[23](#_ENREF_23)]. The 8-LBS performed in previous standing positions (Romberg stand, semi-tandem stand and tandem stand) for a longer time (30 sec instead of 10 sec) and with two different conditions (eyes open, eyes closed). In addition, a fourth standing position (one-leg stand on the preferred leg) is used. This is performed with eyes open, eyes closed, and eyes closed with an additional cognitive distractor. The 8-LBS has a maximum score of 8 points.

The 12-LBS is an extension of the 8-LBS. The one-leg stand on the nonpreferred leg was added and performed with all three conditions (eyes open, eyes closed, eyes closed with an additional cognitive distractor). The 12-LBS has a maximum score of 12 points.

This extension process was performed to avoid ceiling effects, as older employees show a better static balance than do retired seniors.

**Supplementary File 7. Descriptive data for working situation items (n=17)**

| **Variable** | |  |
| --- | --- | --- |
| Professional qualification | n=2 (13%) undergraduate technical college education | |
|  | n=10 (60%) master craftsman/technician degree or another advanced technical college degree | |
|  | n=2 (13%) doctor | |
|  | n=1 (7%) master's degree | |
| Employment status | n=1 (7%) employed temporary | |
|  | n=12 (80%) employed permanent | |
|  | n=1 (7%) civil servant permanent | |
|  | n=1 (7%) self-employed | |
| Overtime per week | 1.44 hour (±1.98 hour) | |
| Model working hours | n=3 (20%) fixed working hours | |
|  | n=10 (67%) flexitime (without/with core time) | |
|  | n=2 (13%) trust working hours (without/with core time) | |
| Organizational structure | n=2 (13%) strongly hierarchical | |
|  | n=8 (53%) rather hierarchical | |
|  | n=2 (13%) neither | |
|  | n=2 (13%) rather flat | |
|  | n=1 (7%) flat | |

*Notes.* Self-developed questionnaire with items regarding working situation-related data. The questionnaire data for two participants were missing.

**Supplementary File 8. Exploratory pre-post measures for objective assessment of low function participants**

| **Objective assessment** | **Pre Score** | **Post Score** | **Effect size^a^** | **p-value^b^** |
| --- | --- | --- | --- | --- |
| **Neuromotor function** |  |  |  |  |
| 12-LBS Scale, Score (n=10) | 7 (1) | 7.0 (0) | r=-1.000 | *p*=.026* |
| CBM Scale, Score (n=7) | 74.3 (1.2) | 80.5 (9.5) | r=-.857 | *p*=.047* |
| **Strength** |  |  |  |  |
| 60CST, number of repetitions (n=7) | 34 (5.5) | 40 (5) | r=-1.000 | *p*=.036* |
| **Physical Activity** |  |  |  |  |
| **Physical related PA** |  |  |  |  |
| Standing time, min (n=7) | 185.8 (23.3) | 165 (46.5) | r=.214 | *p=*.688 |
| Physical activity time, min (n=8) | 91.4 (16.7) | 95.3 (18) | r=-.389 | *p=.*383 |
| Steps, number (n=7) | 7571.8 (1175.5) | 7618.8 (780.4) | r=-.214 | *p=.*688 |
| Sit-to-stand, number (n=7) | 33.2 (6.5) | 38.4 (9.2) | r=-1.000 | *p=.*016* |
| **Sedentary related PA** |  |  |  |  |
| Sedentary time, min (n=7) | 639.2 (20.2) | 613.4 (40.5) | r=.214 | *p=.*688 |
| Sedentary bouts >20min, min (n=7) | 450.8 (71.4) | 399.6 (43.5) | r=.286 | *p=.*578 |
| Sedentary bouts >30min, min (n=7) | 336.2 (93.3) | 333.5 (50.1 | r=.429 | *p=.*375 |
| Sedentary bouts >60min, min (n=7) | 125.5 (99) | 135.8 (22) | r=.143 | *p=.*813 |
| Sedentary bouts >20min, number (n=7) | 9 (1.1) | 9.7 (1.2) | r=-.571 | *p=.*219 |
| Sedentary bouts >30min, number (n=7) | 6.1 (1.7) | 6.1 (1.2) | r=.333 | *p=.*461 |
| Sedentary bouts >60min, number (n=8) | 2.2 (1.3) | 1.6 (0.6) | r=.600 | *p=.*129 |

*Notes. The data are presented as the medians and interquartiles. 12-LBS= 12-Level Balance Scale; CBM=Community balance and mobility scale; 60CST= 60sec chair test.*

**Supplementary File 9. Exploratory pre-post measures for objective assessment**

| **Neuromotor function (12-LBS: n=15, CBM: n=14)** | |
| --- | --- |
| *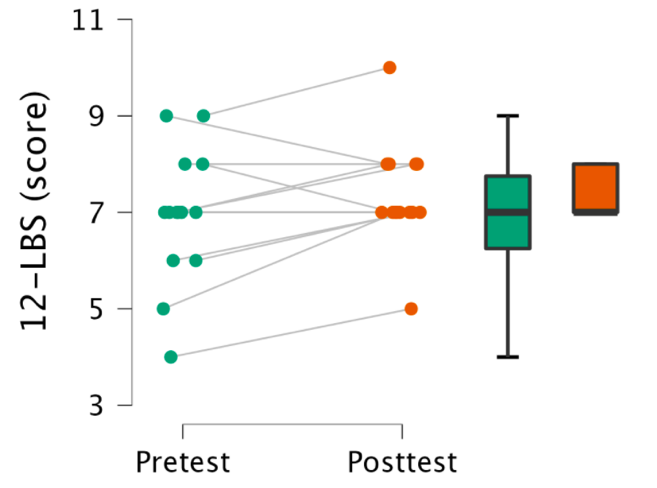* | 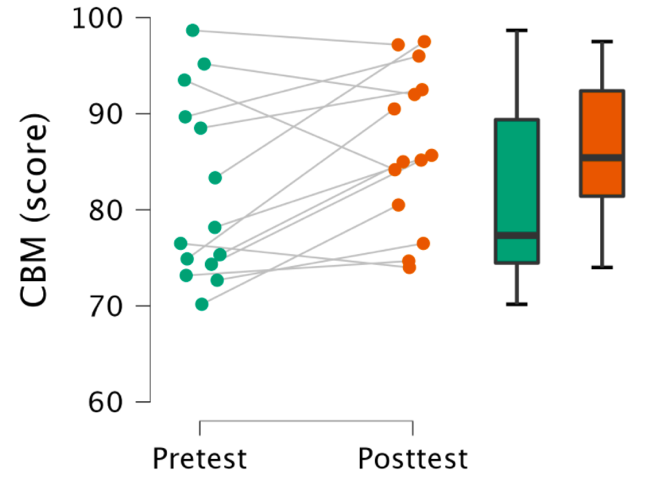 |
| **Strength (n=14)** | |
| 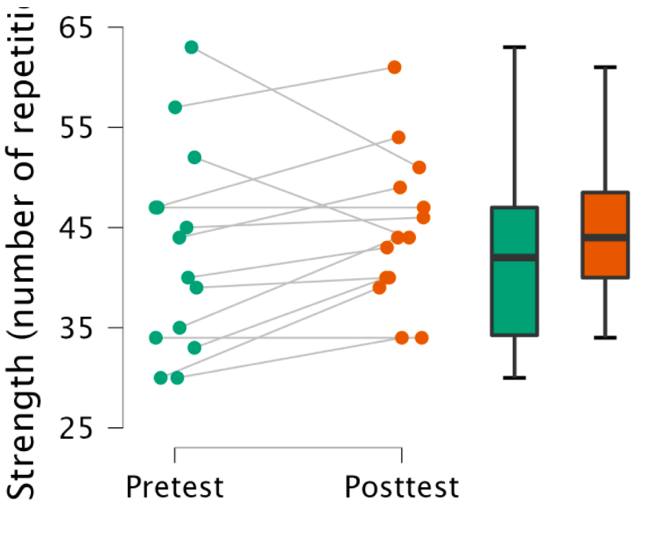 |  |
| **Activity-related PA variables (n=13)** | |
| 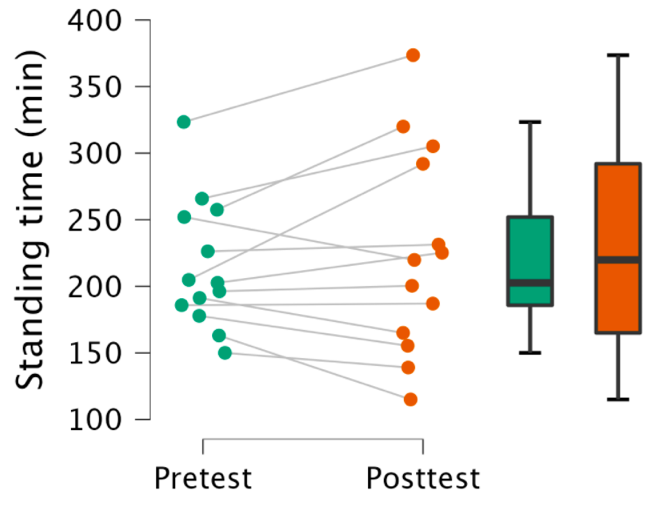 | 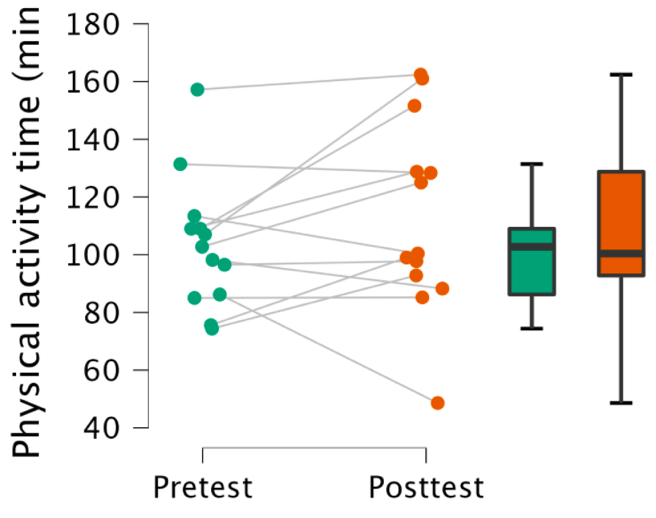 |
| 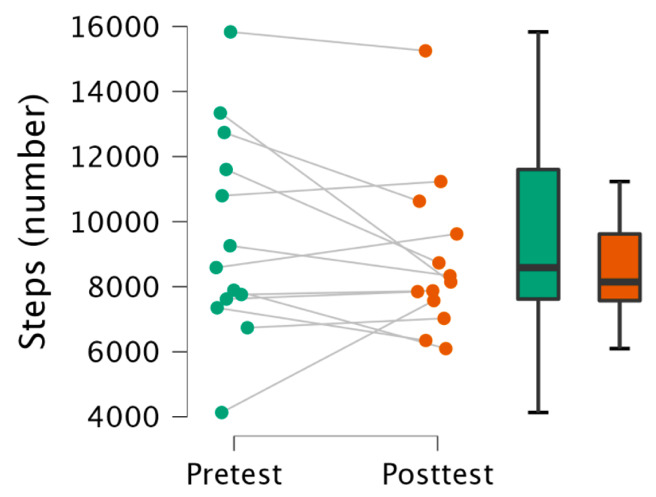 | 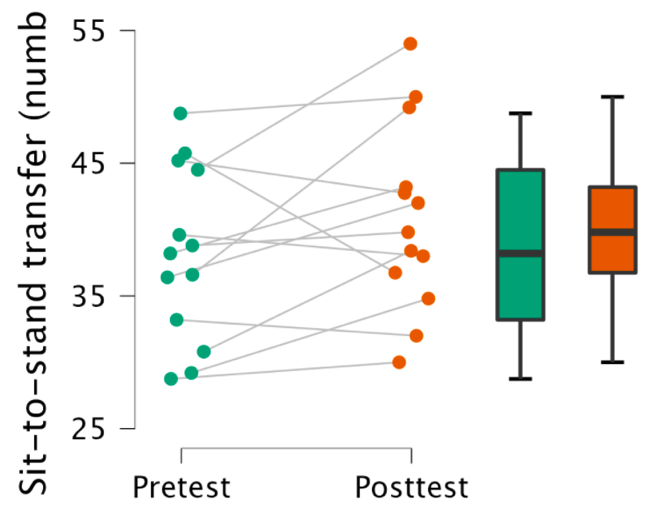 |
| **Sedentary-related PA-variables** | |
| 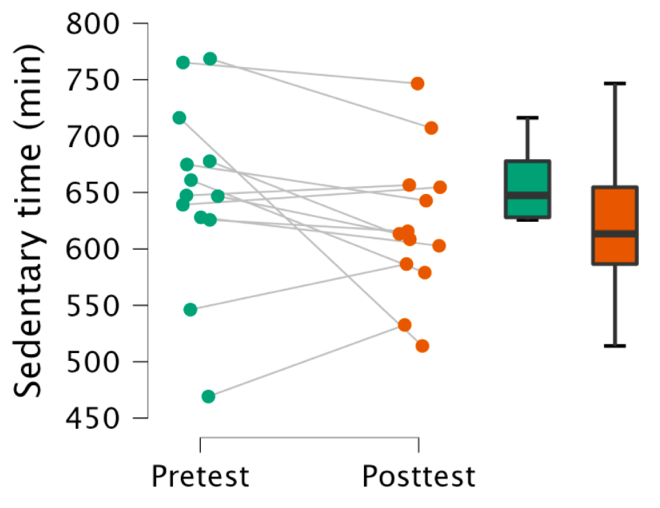 |  |
| 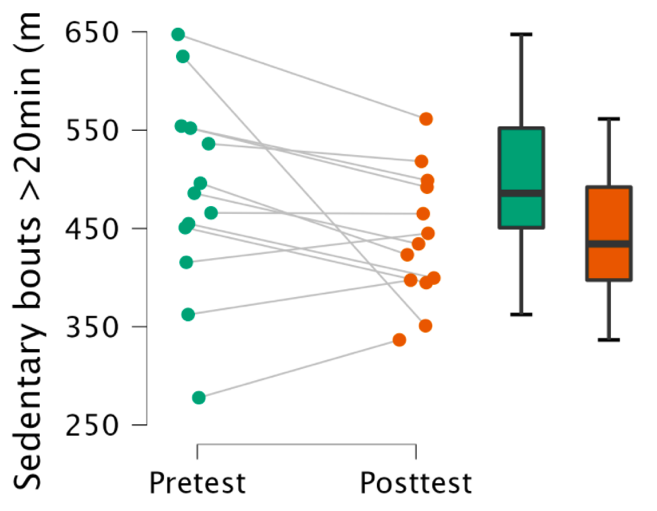 | 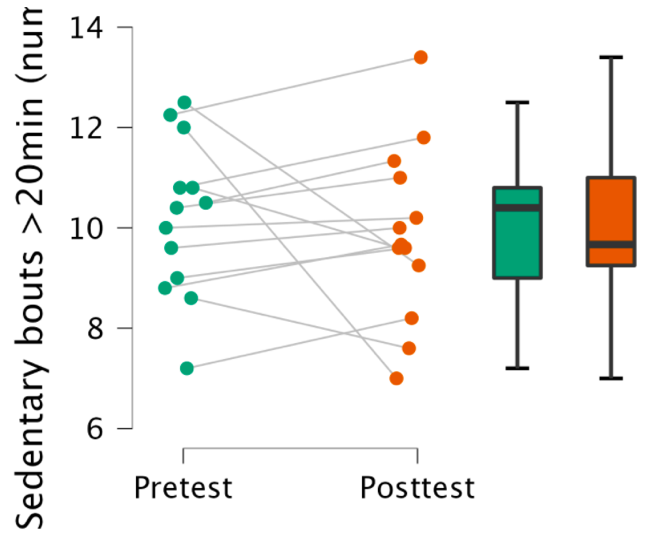 |
| 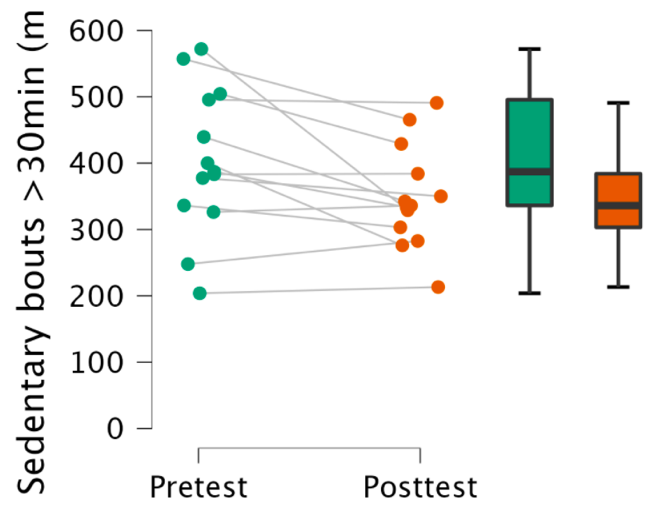 | 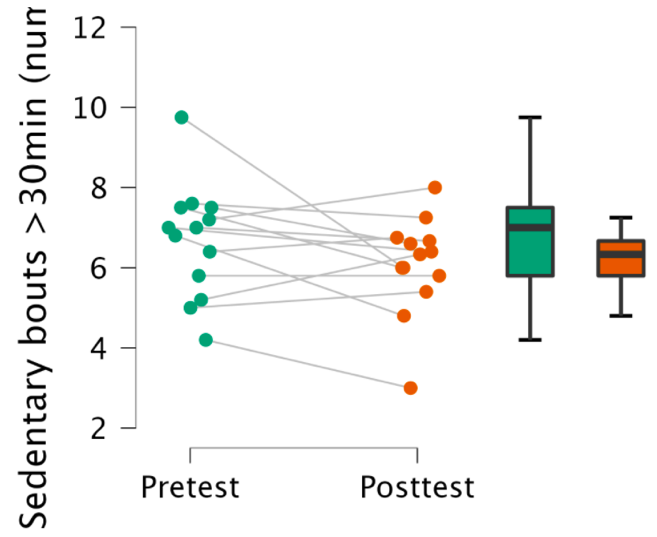 |
| 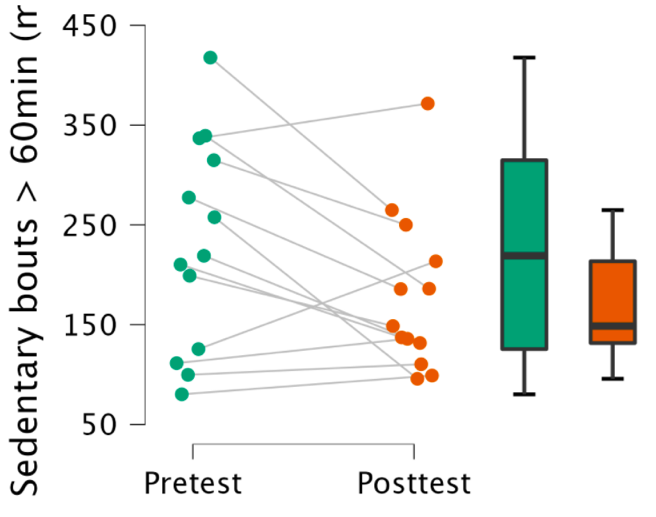 | 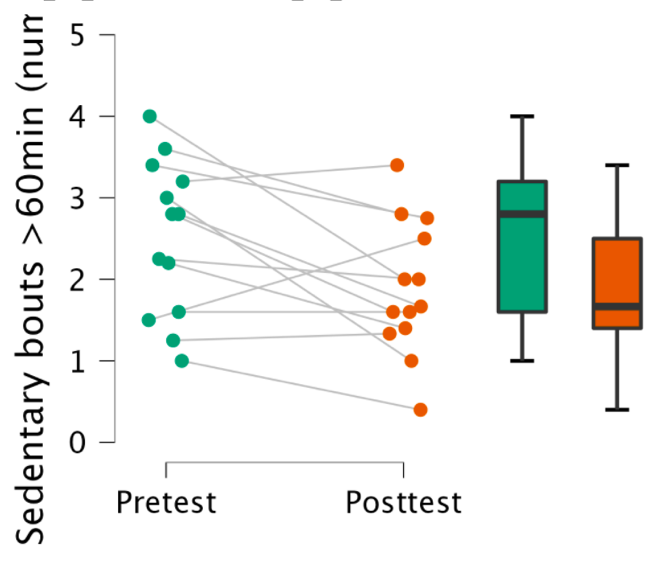 |
